# Y-chromosome phylogeographic analysis of the Greek-Cypriot population reveals elements consistent with Neolithic and Bronze Age settlements

**DOI:** 10.1186/s13323-016-0032-8

**Published:** 2016-02-11

**Authors:** Konstantinos Voskarides, Stéphane Mazières, Despina Hadjipanagi, Julie Di Cristofaro, Anastasia Ignatiou, Charalambos Stefanou, Roy J. King, Peter A. Underhill, Jacques Chiaroni, Constantinos Deltas

**Affiliations:** Molecular Medicine Research Center and Laboratory of Molecular and Medical Genetics, Department of Biological Sciences, University of Cyprus, Kallipoleos 75, 1678 Nicosia, Cyprus; Aix Marseille Université, ADES UMR7268, CNRS, EFS-AM, Marseille, France; Department of Psychiatry and Behavioral Sciences, Stanford University, Stanford, CA USA; Department of Genetics, Stanford University, Stanford, California 94305 USA

**Keywords:** Cyprus, Y-chromosome, Neolithic, Bronze Age, Migration

## Abstract

**Background:**

The archeological record indicates that the permanent settlement of Cyprus began with pioneering agriculturalists circa 11,000 years before present, (*ca.* 11,000 y BP). Subsequent colonization events followed, some recognized regionally. Here, we assess the Y-chromosome structure of Cyprus in context to regional populations and correlate it to phases of prehistoric colonization.

**Results:**

Analysis of haplotypes from 574 samples showed that island-wide substructure was barely significant in a spatial analysis of molecular variance (SAMOVA). However, analyses of molecular variance (AMOVA) of haplogroups using 92 binary markers genotyped in 629 Cypriots revealed that the proportion of variance among the districts was irregularly distributed. Principal component analysis (PCA) revealed potential genetic associations of Greek-Cypriots with neighbor populations. Contrasting haplogroups in the PCA were used as surrogates of parental populations. Admixture analyses suggested that the majority of G2a-P15 and R1b-M269 components were contributed by Anatolia and Levant sources, respectively, while Greece Balkans supplied the majority of E-V13 and J2a-M67. Haplotype-based expansion times were at historical levels suggestive of recent demography.

**Conclusions:**

Analyses of Cypriot haplogroup data are consistent with two stages of prehistoric settlement. E-V13 and E-M34 are widespread, and PCA suggests sourcing them to the Balkans and Levant/Anatolia, respectively. The persistent pre-Greek component is represented by elements of G2-U5(xL30) haplogroups: U5*, PF3147, and L293. J2b-M205 may contribute also to the pre-Greek strata. The majority of R1b-Z2105 lineages occur in both the westernmost and easternmost districts. Distinctively, sub-haplogroup R1b- M589 occurs only in the east. The absence of R1b- M589 lineages in Crete and the Balkans and the presence in Asia Minor are compatible with Late Bronze Age influences from Anatolia rather than from Mycenaean Greeks.

**Electronic supplementary material:**

The online version of this article (doi:10.1186/s13323-016-0032-8) contains supplementary material, which is available to authorized users.

## Background

The island of Cyprus is located *ca.* 100 km from the northern Levant and Anatolia. Evidence from both site excavations and genetics support it being a threshold from which maritime colonists commenced entry to the Mediterranean basin and southeast Europe [1–3] as well as a recipient of different cultural traditions reflecting subsequent human migratory events.

Major phases of prehistoric settlement in Cyprus based on material culture are summarized in Additional file 1: Table S1. In brief, the presence of human activity commenced 13,000 years ago when Mesolithic hunter-gatherers appear in the archeological record [4] followed by colonists associated with Pre-Pottery Neolithic A (PPNA, 11,000–10,400 years before present (y BP)) and B (PPNB, 10,500–8800 y BP) traditions, respectively [4–6]. Subsequent influential episodes of settlement and commerce occurred during the Pottery Neolithic (7200–6000 y BP) and the Early Bronze Age or Philia Horizon (4400–3700 y BP) [4, 7, 8]. The Pottery Neolithic and Early Bronze Age settlements were found in the Northwest/West/South portions of the island [4]. The Late Bronze Age settlements reflected maritime commerce in the Eastern Mediterranean and are concentrated in the East/Northeast regions of Cyprus. This was followed by widespread societal collapse throughout the eastern Mediterranean 3200 years ago [8] and recoveries associated with the Assyrian, Phoenician, Hellenistic, Roman, and Ottoman periods [9].

Some extant Cypriot Y-chromosome data exists but it is either restricted to particular haplogroups [10] or of limited phylogenetic resolution [11, 12]. Considering their language and customs, the Greek element is expected in Greek-Cypriots but little is known about the genetic constitution provided from earlier occupation periods. However, genome-wide studies indicate that the genetic affinity of Cyprus is nearest to current populations of the Levant [2, 13], and an analysis of ancient mitochondrial DNA (mtDNA) from PPNB era associated specimens from Syria identified both U* and K lineages (also present in modern Cypriots) as part of the pre-Bronze context [14]. Here, we report a high-resolution analysis of over 600 Y-chromosomes from contemporary Greek-Cypriots throughout Cyprus, whereby we explore the hypothesis that the present-day male genetic diversity of Cyprus also retains some elements distributed prior to the Hellenic period, with the following objectives in mind: (i) How does the Cypriot population compare genetically with surrounding populations? (ii) Which Y-chromosomes may reflect the Greek versus the pre-Greek settlements of Cyprus?

## Methods

### Sample collection

All aspects of sample selection and anonymity protections were managed by the Greek-Cypriot coauthors at the Molecular Medicine Research Center (MMRC) of the University of Cyprus. The Cyprus National Bioethics Committee approved the research program and the informed consent process. Most samples were collected through organized blood donation activities island-wide. Supplement DNA samples came from the DNA Biobank at the MMRC of the University of Cyprus. Specifically, 105 DNA samples came from the Biobank resource and the remainder from volunteers recruited during blood donations. All donors gave their signed consent for analyzing their DNA anonymously, according to the procedures approved by the Cyprus National Bioethics Committee. Genomic DNA was isolated from the whole peripheral blood [15] from 629 healthy unrelated Greek-Cypriot adult males. We attempted to achieve adequate geographical coverage by sampling from 300 different locations of all six official districts of Cyprus with sample number proportional to population size of each district (Additional file 2: Figure S1). The six districts are Kyreneia, Nicosia, Pafos, Limassol, Larnaka, and Ammochostos (alternate name: Famagusta). Criteria for district membership were continuity of familial origin over at least two generations. Although north Cyprus has been occupied by Turkey since 1974, the northern samples shown in Additional file 2: Figure S1 reflect displaced Greek-Cypriot donors currently living in southern Cyprus but whose familial paternal ancestral heritage traces to the north.

### Genotyping

Genotyping was conducted in both Nicosia and Marseille. Samples were screened hierarchically, using 92 biallelic markers whose specifications are given in Additional file 3: Table S2. The nomenclature used reflects the 2015 version reported in the International Society of Genetic Genealogy (ISOGG) resource, an open science marker aggregator that adheres to the mutation-based rules proposed by the Y Chromosome Consortium (YCC) [16]. All J1-M497-derived chromosomes were categorized on the basis of DYS388 repeat length with ‘long’ being ≥15 repeats [17]. All J2a-M530-derived chromosomes were fractionated on the basis of the DYS445 6, 10, and 11 tandem repeat alleles [18]. The particular nucleotide sequence repeat element counted for the Y-STR loci is given in Additional file 3: Table S2, and the nomenclature used is that of [19].

We genotyped Y-STRs in 574 of the 629 samples, 572 of which pertain to haplogroups (E-M215, G-Page94, J-M304, R-M207) using 17 loci: DYS19, DYS385 a/b, DYS389 I, DYS389 II, DYS390, DYS391, DYS392, DYS393, DYS437, DYS438, DYS439, DYS448, DYS456, DYS458, DYS635 (Y-GATA-C4), and Y-GATA-H4 in the AmpFLSTR® Yfiler® PCR Amplification Kit (Applied Biosystem ® Yfiler, Foster City, USA) following the manufacturer’s instructions. We also genotyped three additional Y-STRs: DYS388, DYS445, and DYS461 in the same 574 samples. A Y-STR based phylogenetic network of E-V13 haplotypes defined by 15 loci was constructed using the program Network 4.6.1.1 (Fluxus-Engineering) with the median joining algorithm. The 15 loci used were DYS19, DYS389 I, DYS389 II, DYS390, DYS391, DYS392, DYS393, DYS437, DYS438, DYS439, DYS448, DYS456, DYS458, DYS635 (Y-GATA-C4), and Y-GATA-H4. For the multi-copy short tandem repeat or microsatellite (STR) DYS389I,II, the DYS389b value was DYS389I subtracted from DYS389II. We also included nine E-V13 haplotypes from Anatolian Greeks—Phokaia and Smyrna [20] for a Greek comparison population.

### Population genetic structure

We examined the population structure within Cyprus by using four approaches: genetic distance, spatial autocorrelation analysis, spatial analysis of molecular variance (SAMOVA), and analysis of molecular variance (AMOVA). First, Nei and Takezaki’s genetic distance [21] between Cypriot Y-STR haplotypes were estimated with Arlequin [22] and plotted by multidimensional scaling (MDS) using *R* [23]. Second, Cypriot Y-STR haplotypes and their geographical coordinates were used to detect possible patterns of isolation by distance within Cyprus through an autocorrelation spatial analysis with even distance classes of 15, 25, and 50 km with the GENALEX software [24]. Third, Y-STR haplotypes and Y haplogroup frequencies were subject to a SAMOVA in order to examine the genetic variance among the six Cypriot districts [25]. SAMOVA implements an objective analysis of the genetic variance to search for geographically homogeneous groups and those differentiated from each other by the highest proportion of variance. We then attempted to identify geographic locations of the sharpest gradients of genetic variation in a manner independent of the six administrative districts by means of a SAMOVA analysis of the STR haplotype data. For this, we partitioned Cyprus into 38 areas of equal sample size of 15 haplotypes. Numbers of areas per district are: Paphos: 7, Ammochostos: 7, Kyreneia: 3, LarnaKa: 4, Limassol: 11, Nicosia: 6. Mean distance between centers of two adjacent areas is 10.09 ±3.93 km. We then ran SAMOVA 2.0 from *K* = 2 to *K* = 10 groups, *K* = 15, and *K* = 20 groups.

Last, Y-chromosome haplogroup frequencies were used to test four historical models through an analysis of molecular variance (AMOVA) [22]. In order to test if the proportion of variance between the different district populations under study was asymmetrically distributed in Cyprus, we started with an AMOVA analysis considering the six populations as one group. We then tested four groupings according to three models of settlement of Cyprus: coastal versus inland (i.e., more than 5 km from the seashore); earliest (Nicosia, Pafos, Limassol) versus Bronze Age occupation sites (Ammochostos, Larnaka, Kyreneia); and two submodels of the arrival of the Philia phase (4400–3700 y BP) with its distinctive pottery style, Red Polished (Ammochostos, Larnaka versus the rest; and Ammochostos, Larnaka versus Kyreneia and the rest) (Table 1 and Additional file 1: Table S1). In order to assess the archeological geographic subdivision between the Pottery Neolithic and Early Bronze Age settlements (densely populating Northwest/West/South Cyprus) from the Late Bronze Age settlements (East/Northeast Cyprus), we a priori divided the island into two regions: (1) Kyreneia/Nicosia/Pafos/Limassol versus (2) Ammochostos/Larnaka and performed 2 × 2 table chi-square comparisons of frequency distributions of specific Y-chromosome haplogroups.

**Table 1:** SAMOVA and AMOVA among the six district of Cyprus based on Y-STR haplotypes and haplogroup frequencies (Hgp freq)

Geographical structure	No. of groups	Percent of variation			
		Within population	Among populations within groups	Among groups	
Departing districts from the rest of Cyprus		Hg freq	Hg freq	Y-STRs	Hg freq
Kyreneia	2			8.27	
Kyreneia. Ammochostos	3			6.01	
Kyreneia. Ammochostos. Limassol	4			4.68*	
Larnaka/Nicosia	5			4.08	
Kyreneia	2				0.57
Kyreneia. Larnaka/Ammochostos	3				0.39*
Kyreneia. Ammochostos. Pafos	4				0.49*
Limassol/Nicosia	5				0.66**
Cyprus. 6 districts as a whole	1	99.39	0.61***		–
Coast. inland	2	99.38	0.53**		0.09
(Nicosia. Pafos. Limassol). (Kyreneia. Ammochostos. Larnaka)	2	99.32	0.51***		0.18
(Kyreneia). (Nicosia. Pafos. Limassol). (Ammochostos. Larnaka)	3	99.25	0.36*		0.39*
(Larnaka. Ammochostos). (Kyreneia. Limassol. Nicosia. Pafos)	2	99.30	0.51**		0.19

*p < 0.05; **p < 0.01; ***p < 0.001

### Population relationships

To assess Cyprus in a broader regional context, we gathered Y-chromosome haplogroup frequencies from 36 populations totaling 4666 males, from relevant surrounding regions, Levant-Middle East (Lebanon, Iraq/Baghdad, Egypt, Asia Minor), Caucasus (Armenia), continental Balkans (Bulgaria, Albania, Croatia), Mediterranean northern coast (Italy, Greece), Central Europe (Czech Republic, Hungary), and Mediterranean Islands (Crete, Sicily, Sardinia, present study) [12, 18, 26–32]. Since the various studies differed in regards to haplogroup resolution, we standardized the data to 25 haplogroups as well as appropriately collapsing the Cypriot data (Additional file 4: Table S3).

We then investigated the genetic affinities among the populations by principal component analyses (PCA) using XLSTAT 7.5.2.

### Admixture contribution to Cyprus

Based on the pre- and historical events in Cyprus (Additional file 1: Table S1), we attempted to measure the genetic contribution of Anatolian, Balkans, Greek, and Levantine parental populations to Cyprus. Towards this aim, we first used Y-chromosome haplogroup frequencies from Additional file 4: Table S3 to measure the *mY* estimators with 100 replications as implemented in ADMIX 2.0 [33]. We then focused on E-V13, G-P15, I2-M423, J2a-M67, J2b-M12, and R1b-M269 haplogroups, that we view as the most appropriate Anatolian, Balkan (Danube basin, Greece), and Levant proxies reflecting Neolithic and Bronze Age human dispersals (e.g., [18, 30, 31, 34, 35]). We compiled lineage (i.e., coupled single-nucleotide polymorphism (SNP) and STR) data for these surrogates from [10, 12, 18, 20, 28, 31, 35–40] and performed ADMIX95 [41] to measure the *m* estimators of Anatolia, Danube Balkans, Greece, and Levant to the six fore mentioned Cypriot haplogroups. The lineages were composed of 11 loci (DYS19, DYS388, DYS389I, DYS389B, DYS390, DYS391, DYS392, DYS393, DYS437, DYS438, and DYS439). All input files for both ADMIX packages were built using the AdFiT v1.7 tool [42]. A high *R*^2^ means that the allelic frequencies in the hybrid population can be explained by the allelic frequencies in each of the parental population.

### Date estimates

We estimated the time of expansion (TIMEX) of E-V13, G2a-P15, I2-M423, J2a-M67, J2b-M12, and R1b-M269 lineages in Cyprus and their time of divergence (TD) from Anatolian, Balkans, Greek, and Levantine sources. TIMEX and TD date lineages assuming that the observed variance has arisen indigenously within a given population as a result of a unique founder-based migration from an outside source. In practice, this means measuring the variation between the source and sink population assuming that it has accumulated since the time of settlement.

For TIMEX, variation was measured from the mean variance of the abovementioned 11 microsatellites. TD was based on the square difference between the means of allele size incorporated in the genetic distance denoted (δ*μ*)^2^ [43] and implemented in POWERMARKER [44]. Under the assumption of a single population splitting into two fully isolated groups, this genetic distance is supposed to increase linearly with time since divergence as (δ*μ*)^2^ = 2*ωτ*, where *ω* is the mutation rate and *τ* is the number of generations since isolation. Note that these approaches are sensitive to both multiple founders during a particular migration as well as subsequent population gene flows, both of which inflate STR variance and the age estimate of the event. Since the choice of mutation is debatable (e.g., [45, 46]), we used two mutation rates for STR: 0.00069 per STR per generation [47] and pedigree mutation rate of 0.0021 with 95 % confidence interval limit (CIL) of 0.0006–0.0049 % [48] to set lower and upper bounds. We assumed a generation time of 25 years.

## Results

### The complex genetic structure of Cyprus

**Fig. 1:**
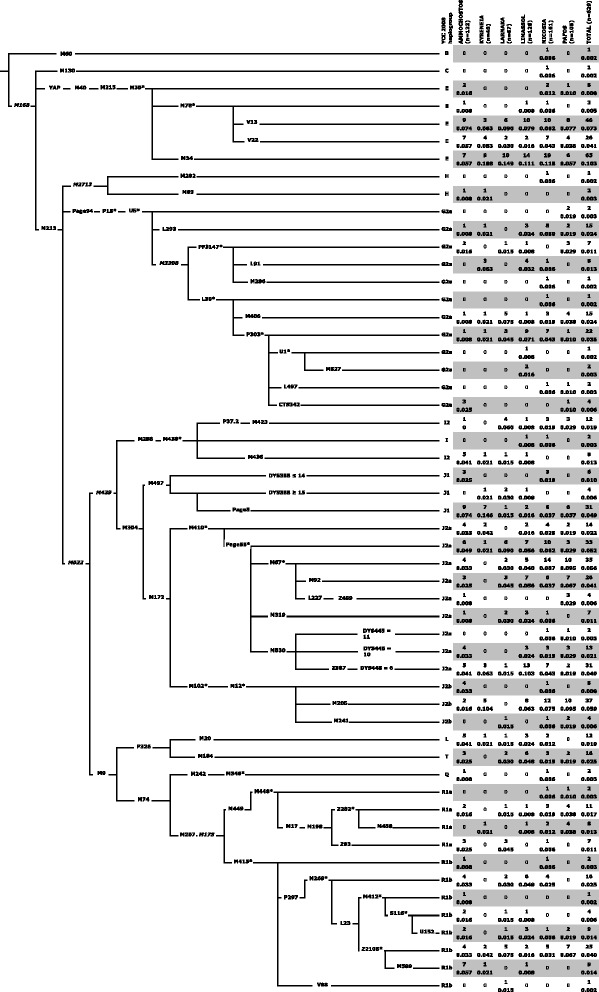
Topological relationship of Y-chromosome binary markers and their observed haplogroup frequencies (absolute and relative) in the six districts of Cyprus. Nomenclature used in that recommended by [89]. Common names of markers are shown along the branches whose lengths are uninformative with respect to time. The *asterisk* refers to the unresolved status of the paragroup beyond the specific polymorphism. Six markers shown in *italic font* were not genotyped but provide context. The following 18 binary markers (with their haplogroup affiliation) were also genotyped but displayed no derived alleles: (E) V42, V6, V92, V257, M81, (G) P20, P16.1, P16.2, (I) M26, (J2a) M318, M419, M322, (O) M175, (Q) M25, M3, M378, (R2) M479, and (R1b) U106

Figure 1 presents the phylogenetic relationships and frequencies of the Y-chromosome lineages detected in the six districts of Cyprus. Like other populations in Anatolia and Lebanon, Cyprus exhibits a high level of haplogroup J2-M172 related diversity. J2a-M410 is the dominant Y-chromosome lineage, constituting 26.0 % of the overall Cypriot samples. J2b-M12/M102 splits into mainly J2b-M205 (5.9 %), frequent in Southern Levant (Additional file 5: Figure S2), and J2b-M241 (0.6 %), most frequent in Greece and the Balkans [20, 35]. Overall, the E-M35 haplogroup totals to 23.1 % and contains various E-M78 sub-haplogroups including E-V13 (7.3 %) that is common in Greece [10, 18, 35] and E-V22 (3.5 %), that is frequent in Egypt [10] and Sudan [49]. Another E-M35 related haplogroup, E-M34, previously reported in Asia Minor [31], Southern Levant [50, 51], and the Balkans [35] also was observed in Cyprus (10.3 %).

In Cyprus, haplogroup G2a-U5 (12.9 %) is widely distributed. While only 0.3 % distribute to the U5* paragroup, the PF3147 component (2.5 %) includes lineages like L91 (1.3 %), also seen in Asia Minor and Crete [30] and attributed to reflect the early Neolithic settlement of Sardinia [52]. The G2a-L293 lineage emerges directly from the U5 node and occurs at 2.4 %, a level similar to that observed in Anatolia and the Levant (Additional file 5: Figure S2). Within the L30 defined clade (7.5 %), all lineages with the exception of L497 also occur in Anatolia [30] including the L30* paragroup (0.2 %). Haplogroup G2a-M406 (2.4 %) occurs at similar levels in Asia Minor and also on Crete (1.9 %), one of the earliest (*ca.* 9000 y BP) known sites outside the Levant colonized by Neolithic peoples [18]. Within the G2a-P303 portion (4.9 %), the as yet unresolved P303* paragroup is seen (3.5 %) with both the Central European and Northern Italian G2a-L497 and the G2a-M527 lineages occurring at 0.3 %. The overall frequency, the haplogroup G2a-P303, especially the U1 branch is highest (20–39 %) in populations of the southern and northwest Caucasus [30]. Lastly, we detected G2a-CTS342 (0.6 %), a lineage that has been reported in Sardinia [52] and as well in ancient DNA from Asia Minor [53].

Unlike samples from the present day interior Levant, such as Palestinians, Bedouins, and Jordanians [17, 54], J1-M267 is less common in Cyprus at 6.5 %. Haplogroup I2a lineages, thought to have arisen from a post-Last Glacial Maximum refuge and now present in Balkans, Sardinia, and Northwestern continental Europe [35, 55], were observed at *ca.* 3.5 % in our sample. Overall, haplogroup R1 presence was 15.1 %. The total frequency of associated R1a-M449 and R1b-M415 sub-haplogroups was 4.5 % and 10.7 %, respectively. The paragroup R1b-M269*(xL23) lineage is present (2.5 %). Furthermore sub-branches reflecting distinctive European versus Asian divergences similarly occur in both R1a and R1b. Within R1b, the central/west Europe M412 constituent (2.2 %) is offset by the western/central/south Asia Z2105 fraction (5.4 %) that was previously reported as paragroup L23*(xM412) [56]. Similarly in R1a, both the European Z282 component (3.0 %) and the counterpart Asian Z93 clade (1.1 %) occur. Notable is finding that none of the R1a-Z93 Cypriots carried the diagnostic Ashkenazi Levite DYS456 14 repeat YSTR allele [57]. Lastly, traces of geographically remote B-M60, C-M130, L-M20, and Q-M346 lineages were detected, mainly in the Nicosia district.

### Island substructure

Spatial analysis of Y-STR variance among the six Cypriot districts barely showed significant geographical structure at *K* = 4 with northern, eastern, and southern regions separating (4.68 %, *p* < 0.05, Table 1). The nearly featureless geographic structure of the haplotype data is reiterated by the non-significant spatial autocorrelation (Additional file 6: Figure S3) as well as a lack of genetic affinity with district affiliation displayed in the MDS plot (Additional file 7: Figure S4). On the contrary, dispersal of Y-chromosome haplogroups (Fig. 3) reached significance at *K* = 3 to 5 (range of percentage of variation, 0.39–0.66 %, *p* values <0.01, Table 1).

We then searched for shared STR haplotypes .While the majority of our 574 STR haplotypes are unique we observed 197 perfect matches, most of which are shared within the same district and to a lesser extent between adjacent districts, indicative of recent demographic growth. To assess the degree of possible local patterns of genetic diversity we conducted a SAMOVA using the distributions of 574 hts within a uniform grid of 38 areas across the island. Mainly single areas separated first (Additional file 8: Table S4). However at the K= 3 level (Fig. 3) we detected two clusters that separate from the remaining samples, one of which is composed of 3 coastal grid areas that is characterized by 17.8 % of J2a-M67 derived chromosomes. The other cluster, comprised of two grid areas in the center of the island, entirely lacks any J2a-M67. At higher levels, K=6 and above, significant zones of reduced variance are restricted to single grid areas indicative of recent growth.

**Fig. 2:**
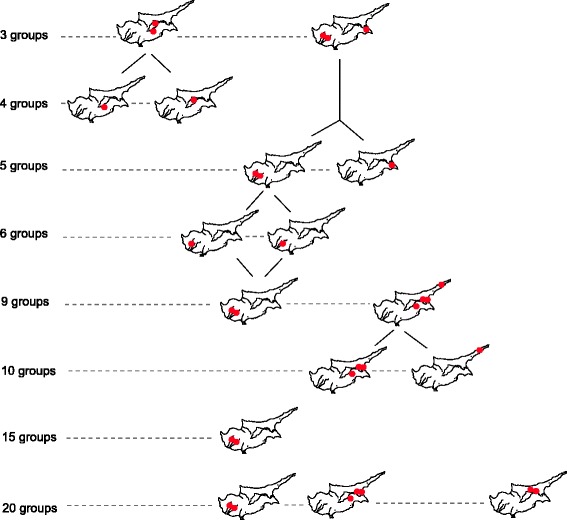
Location of Y-STR haplotypes grouped by SAMOVA and their evolution from *K* groups to *K* + 1. Proportion of variance between groups ranged from 10.39 to 15.87 (*p* < 0.0001)

To assess the degree of possible local patterns of genetic diversity shaped by recent demographic forces, we conducted a SAMOVA using the distributions of 574 haplotypes within a uniform grid across the island. From *K* to *K* + 1, single areas stood out from the rest of Cyprus (Additional file 8: Table S4). Wherever we detected statistically significant zones of reduced variance (red dots in Fig. 2, Additional file 8: Table S4), the amount of within-group variance made them split at *K* + 1. Small geographical patterns of reduced genetic variation remained in the Southwest and in the East, attributable to recent demography processes associated with genetic drift. Before we tested for any possible signals of correspondences between settlement history and predefined groups of districts, we first confirmed that male lineage distribution among the six Cypriot districts (Table 1) showed an uneven percentage of variation (0.61 %, *p* value <0.001). The inland/coast partition model did not significantly pull apart the variance (0.09 %, *p* value >0.05). Neither the Central South West versus East partition (Pottery Neolithic to Early Bronze Age versus Late Bronze Age occupation site model) nor the East versus the rest of Cyprus (one of the Philia submodels) were found significant (0.18 and 0.19, respectively, *p* > 0.05). However, the best clustering with a district was found for one of the two submodels concerning the arrival of the Philia phase, namely the Pottery Neolithic to Early Bronze versus Late Bronze Age occupation site model, when Kyreneia is considered separately (0.39 %, *p* value <0.05).

**Fig. 3:**
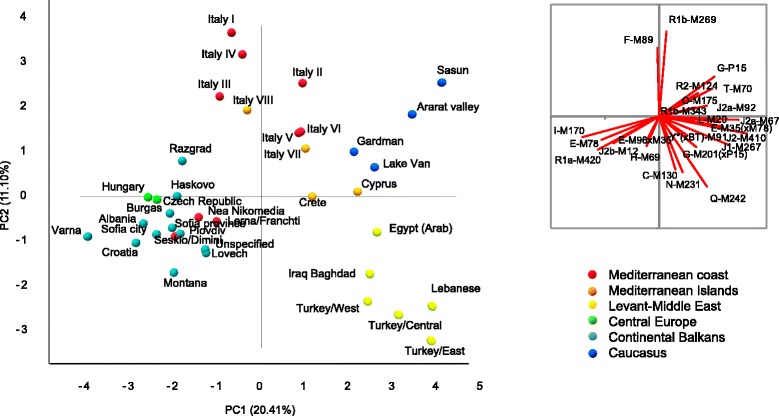
Plot of the two first coordinates from principal component analysis depicting the relationship of Cypriot with 35 other regional populations based on frequencies of 25 comparable Y-chromosome haplogroups as described in the “Methods” section. The separate vector image reflects the role of various haplogroups with the population distributions

PCA shown in Fig. 3 depicts the main genetic relationships between Cyprus and surrounding populations based on Y-chromosome haplogroup frequencies. Axes 1 and 2 contribute 31.5 % of the total variance. The distributions of the populations trend with geography (*R*^2^ Pearson’s coefficient of correlation with latitude and longitude above 0.092, *p* < 0.05). On axis 1, Bulgarian, Czech, Balkan, Hungarian, and Greek groups stand apart from Caucasus and Near Eastern populations, while axis 2 separates the Italian and Near Eastern groups. Notably, Cyprus and Crete occupy a central position. Caucasus, southern Italian, Crete, and neighbor Arab-speaking Egyptian and Iraqi populations show closer genetic relationships with our Cypriot sample set. A recent autosomal survey also revealed genetic affinity between Cypriot and Caucasus individuals, probably dating back from the early Bronze Age [13]. Noticeably, the long-term affiliated closest population, mainland Greeks, is genetically more distant than the aforementioned populations, clustering rather with the Balkan and Bulgarian groups. Such similarity between Greece and northern Balkans could trace back to the emergence of the Starcevo culture [58] in early Neolithic (8500 y BP).

### Ancestry of male Cypriot lineages

Comparing the entire set of Y-chromosome haplogroups with those from regional populations surrounding Cyprus revealed a high Anatolian influence (*mY* = 66 %), followed by the Levant (*mY* = 24 %) then the Balkan regions (*mY* = 13 %, Table 2). A putative Roman contribution to Cyprus using data from Italy and Sicily [32] also showed negative values of *mY* (data not shown). A closer look at specific proxy lineages permitted us to dissect these results further (Table 2). Anatolia would have generated up to 83 % to the Cypriot G-P15 and up to a quarter (range, 22–25 %) of Cypriot J2a-M67, J2b-M12, and R1b-M269 related lineages. Danube Balkans would have provided most of the Cypriot J2b-M12 (67 %) and all Cypriot I2-M423 (99 %). Although, when using the entire set of Y-chromosome haplogroup frequencies, the composition of Cyprus can be explained by contributions from Anatolia, Balkans, and Levant, the actual Greek contribution stood out for the Cypriot E-V13 (87 %), J2a-M67 (74 %), R1b-M269 (48 %), and G-P15 (17 %) components. Lastly, Levant contributed up to 30 % of the Cypriot R1b-M269 and to a lesser extent regarding the Cypriot J lineages (3–8 %).

**Table 2:** Bootstrapped (100 iterations) *mY* and *m* estimators of admixture proportions of four parental populations to Cyprus

Haplogroup	Anatolia	Balkans^a^	Danube	Greece	Levant	
	*mY* (s.d.)	*mY* (s.d.)	*mY* (s.d.)	*mY* (s.d.)	*mY* (s.d.)	
All	0.66 (0.05)	0.13 (0.03)	0.10 (0.02)	–	0.24 (0.05)	
	m (s.e.)		m (s.e.)	m (s.e.)	m (s.e.)	*R* ^2^
E-V13	0.13 (0.01)		–	0.87 (0.01)	–	0.9997
G-P15	0.83 (0.07)		–	0.17 (0.07)	–	0.9500
I-M423	0.01 (0.00)		0.99 (0.00)	–	–	0.9998
J2b-M12	0.25 (0.03)		0.67 (0.05)	–	0.08 (0.05)	0.8779
J2a-M67	0.23 (0.03)		–	0.74 (0.14)	0.03 (0.12)	0.9896
R1b-M269	0.22 (0.03)		–	0.48 (0.13)	0.30 (0.15)	0.9897

*s.d.* standard deviation, *s.e.* standard error

^a^Balkans merges Danube with Greece

The pattern of structural variation in Cyprus points towards a model comprising two stages of expansion: an earlier expansion of G2a-P15, J2a-M67, and R1b-M269 (range, 11,600–13,800 y BP with a slow YSTR mutation rate *ω*; 3800–4500 BP with a fast *ω*), subsequently followed later by the expansion of E-V13, I2-M423, and J2b-M12 (slow *ω*, 4400–6600 y BP; fast *ω* 1500–4500 y BP) (Table 3). However, times of divergence of these lineages from current Anatolian, Danubian, Greek, and Levantine Y-STRs appeared more recent. Pre-historical divergence was observed for Cypriot G-P15 with Greece (3600 y BP), I2-M423 with Anatolia (4200 y BP) and Levant (9400 y BP) and J2b-M12 with Danube Balkans (3,500 y BP) and Levant (5100 y BP). Divergence of E-V13, J2a-M67, and R1b-M269 would have taken place in modern times (range, 300–2.200 y BP). Note that both M67 and M269 encapsulate high genetic variance, respectively, 0.352 and 0.320, but little genetic differentiation with Anatolia, Danube Balkans, Greece, and Levant. This suggests either an arrival of multiple diverse founders during the Bronze Age period or alternatively several subsequent flows from these regions. In addition, the use of a fast YSTRS mutation rate reduced the divergence to the historical era. While these YSTR-based estimates are consistent with two stages of settlement, the chronological framework regarding such expansions remains in doubt due to uncertainty regarding the appropriate average YSTR mutation rate for the 11 loci that compose the haplotype.

**Table 3:** Variance, time of expansion (TIMEX in y BP), and time of divergence (TD in y BP) of six major male lineages in Cyprus as compared with Anatolian, Balkan, Greek, and Levantine genetic contributors

		TIMEX Cyprus			TD Anatolia		TD Danube Balkans		TD Greece		TD Levant	
		Mutation rate	0.00069 (s.d.)	0.0021 [95 % CIL]	0.00069 (s.d.)	0.0021 [95 % CIL]	0.00069 (s.d.)	0.0021 [95 % CIL]	0.00069 (s.d.)	0.0021 [95 % CIL]	0.00069 (s.d.)	0.0021 [95 % CIL]
	Variance											
E-V13	0.157		5685 (1071)	1868 [801–6538]	728 (137)	239 [102–837]	422 (80)	139 [59–485]	522 (98)	172 [74–600]	–	–
G-P15	0.38		13758 (2592)	4520 [1937–15821]	1526 (287)	501 [215–1755]	–	–	3641 (686)	1196 [513–4187]	–	–
I-M423	0.182		6588 (1241)	2165 [928–7576]	4213 (794)	1384 [593–4845]	1417 (267)	465 [199–1629]	1648 (310)	541 [232–1895]	9397 (1770)	3087 [1323–10806]
J-M12	0.123		4445 (838)	1461 [626–5112]	1859 (350)	611 [262–2138]	3525 (664)	1158 [496–4054]	–	–	5130 (967)	1686 [722–5900]
J-M67	0.352		12767 (2405)	4195 [1798–14682]	294 (55)	97 [41–339]	1010 (190)	332 [142–1161]	469 (88)	154 [66–540]	670 (126)	220 [94–771]
R-M269	0.32		11590 (2184)	3808 [1632–13329]	2202 (415)	724 [310–2533]	890 (168)	292 [125–1024]	352 (66)	116 [50–405]	528 (100)	174 [74–608]

*s.d.* standard deviation

## Discussion

### Early and subsequent haplogroup dispersals

The strong correspondence between geography and Y-chromosome binary haplogroups is well known [59]. This feature is consistent with a link between the distribution of haplogroups and past human movements. However, the task of deconvoluting prehistoric gene flows from subsequent transformations owing to ensuing migrations, local differentiations, and recent demographic growth (e.g., [60]) overlaid upon previous ones is complicated. For example, more recent migrations may also contain older haplogroups. Despite rigorous geographically targeted sampling and time parallels with cultural traits [20], current attempts to link of modern Y-chromosome patterns to prehistoric events are preliminary and best viewed with prudence. Such interpretations will be reappraised in the future using a combination of approaches, including simulation modeling [61], ancient DNA (e.g., [53, 58, 62]), and assessment of haplogroups that coalesce near the time frames of interest. This latter strategy is plausible due to the development of elaborately branched SNP dense phylogenies with branches proportional to time [63–67]. While, for reasons summarized in [63], ambiguity currently exists regarding an established mutation rate to use for calibration, this uncertainty will narrow as additional pedigree and clan based “whole” single-copy X-degenerate sequences are analyzed [46, 68] and cross-checked by ancient DNA-based rate estimates (e.g., [69]). With these caveats in mind, we proceed to define the putative prehistoric roots of Cypriot male genetic diversity by: (a) identifying lineages representative of non-Greek genetic influences, (b) reporting statistical support for correlations between settlement zones and haplogroup frequencies, (c) taking guidance from preliminary temporal estimates reported in vanguard studies of Y-chromosome phylogenies with meaningful branch lengths (e.g., [63, 64, 66]), and (d) noting coherence of ancient DNA data with pertinent archeological context.

### Genetic legacy/substratum of the aceramic Neolithic

The Neolithic transition has diffused a wide array of culture, economic strategies, and social changes spanning the Levant, the Caucasus, and Europe [70–72] including Mediterranean islands that served as both way stations and terminal settlements [3, 14, 72–74]. Previous Y-chromosome studies [30, 31, 34, 37, 75, 76] hypothesized that lineages from haplogroups G, J, E, and R1b-M269 would have accompanied this cultural expansion although high levels of I2 and E-V13 versus low levels of extant G and J in the Balkans raise the possibility that only vestiges of pioneering agriculturalists to southeast Europe remain [35]. In Europe, certain sub-haplogroups of G and specifically E-V13 were detected in ancient DNA, including Linear Band Keramik (LBK) remains from Central Europe (*ca.* 8000 y BP), Epicardial skeletons from Iberia (7000 y BP), South of France Late Neolithic (5000 y BP), and a Tyrol specimen (5300 y BP) [77–80].

In Cyprus, the G2a-U5 assemblage has an overall frequency of 12.9 %. Notably, PF3147 and related lineages occur mostly in the insular Mediterranean and display frequencies consistent with a relic distribution (e.g., [80]). Haplogroup G-M406 (2.4 %) is widely distributed across the island, but highest near Khirokitia, an aceramic Neolithic site (Table 1) located on the southern coast of Larnaka district. Interestingly, the more deeply rooted sub-haplogroup G2a-L293 also occurs in Anatolia and northern Levant (Additional file 5: Figure S2), consistent with the PPNB crescent including Syrian areas whose maternal genetic legacy is coherent with the maritime movements of early farmers [14]. In addition, recent archeological studies have demonstrated that Epipaleolithic hunter-fisher-foragers colonized the island first (11,000–13,000 y BP) [81, 82], and that these forays are located on the southern coast as well (Aetokremnos, Amathus, Klimonas, and Asprokremnos), on the opposite slope of the Troodos Mountains [6, 83, 84]. Considered together the lithic industry, chronology and locations of these first human settlements match the PPNA tradition from Levant [6]. Cyprus would thus represent one of the first stops of this diffusion, bringing probably some G male lineages to continental and insular Europe [6, 81]. As far as J, E, and R are concerned, refined investigation of their derived lineages could match with further events, characterized by the intensification of commercial exchanges throughout the Levantine Sea and other regions discussed below.

### Centripetal gene flows during pottery Neolithic

It has been hypothesized that J2b-M12 may have been associated with the Neolithic immigration of farmers to Greece [18]. Haplogroup J2b-M12 splits into J2b-M205 and J2b-M241. Since J2b-M241 frequency distribution was already well characterized [35], we mapped the frequency distribution of J2b-M205 (Additional file 5: Figure S2).

Both genetic data from the literature and Syria (Chiaroni J, unpublished results) show a frequency peak of J2b-M205 in the Southern Levant in which the frequency decreases northwards with latitude (Pearson’s *R*^2^ = 0.282, *p* value = 0.011). The J2b-M205 distribution coincides with the Neolithic crop package dissemination found in common between the Levant and Cyprus [5]. Also J2b-M12 Td estimates (Table 3) coupled with the J2b-M205 distribution overlap significantly with the Pottery Neolithic to Early Bronze Age pattern of settlements in Nicosia, Pafos, Limassol, and Kyreneia (chi-square = 11.29, *p* < .00084). This suggests the possibility that Cyprus experienced a later (Pottery Neolithic) immigration from the Southern Levant.

Regarding J2a-M410, the most common M530 sublineages are J2a-Z387 (4.9 %) linked with the distinctive six repeat allele at the DYS445 short tandem repeat locus proposed [18] to represent a Neolithic expansion from Anatolia to Greece and Italy, a pattern similar to G2a-M406 [30], at 4.9 % and J2a-Page55*(xM67,M319,Z387) at 5.2 %. The J2a-M319 lineage, previously observed in Crete and the Levant [18, 85] is also present in Cyprus at 1.1 %. However, its Y-STR haplotype diversity (Additional file 9: Table S5) is considerably higher from that in Crete (variance, 0.279 versus 0.121).

### Early Bronze Age: in search of metals

Recent insights from ancient DNA studies suggest the spread of genes during the Bronze Age involved J2a-M67 individuals who appear in the Central European plains during the Late Bronze Age [86]. J2a-M67, proposed to represent both the Neolithic of Central Anatolia and the expansion of the Troia Maritime Culture in Northwestern Anatolia (13.5 %) [31], is also quite common in Cyprus (10.1 %). Testing the hypothesis that the origin of the Early Bronze Philia culture in Cyprus derives from Western/Northwest Anatolia, the distribution of J2a- M67 on Cyprus fits well (chi-square = 3.42, *p* < .032, one-tailed). J2a-Z489, present in Pafos and Northwest/Central Anatolia, may reflect Bronze Age immigration from Western Anatolia, the Philia phase, or mirror the Jewish population on the island from the Hellenistic/Roman Eras.

### Late Bronze Age Cyprus and maritime trade

E-V13 is common in the Balkans and may mark some of the Greek demographic input to Cyprus from the Late Bronze Age through the Iron Age [79]. Network analysis of 46 E-V13 haplotypes (Additional file 10: Figure S5) shows a discrete clustering of 15 samples suggestive of a sub-haplogroup (encircled with an oval). This cluster is characterized by DYS437 = 15 repeats not seen in the Anatolian Greek population, or in the Provence samples [20]. The remaining 31 samples overlap with the Anatolian Greek E-V13 lineages. Given that the highest frequency of I2 is in the Balkans [28], we also propose that I2-M423 (1.9 %) and I2-M436 (1.3 %) lineages reflect Greek influence. Additionally, the presence of G2a-M527 and G2a-U1 is consistent with remnants of Greek heritage [30]. E-V22 and E-M34 are common in the Southern Levant, Sicily, Algeria, and in Egypt and rare in Europe [27, 36]. These lineages, like J2b-M205, could mirror a Pottery Neolithic movement to Cyprus from the Southern Levant (Pearson *R*^2^ coefficient of correlation of E- M34 to longitude: 0.164, *p* = 0.003).

Haplogroup R1b-L23 membership on Cyprus is predominantly R1b-Z2105 and parallels the M412 lineage that is prolific in central and west Europe [56]. Whole Y-chromosome phylogeny-based estimates of the coalescent times for the M412 and Z2105 companion lineages (and/or their phylogenetic equivalents or nearest neighbors) are reported in the range of 5000–6000 years [63, 64, 87]. While these dates should be viewed as preliminary as discussed previously, all estimates postdate the earlier dates of the aceramic and early ceramic phases of Neolithic settlements. Further underpinning the inference that R1b-Z2105 lineages are plausibly associated with Bronze Age settlement is that the oldest R1b-Z2105 designated lineages detected in ancient DNA specimens occur in the steppe belt regions of Russia and radiometrically date to *ca.* 5000 years ago [58]. In Cyprus, Z2105 has the opposite distribution from J2a-M67 and J2b-M205, concentrating in the East/Northeast regions of Cyprus (chi-square = 5.01, *p* < .0256). The Late Bronze Age cities of Enkomi, Kition, and Hala Sultan Tekke, found in the Larnaka and Ammochostos districts, may have received immigrants from Hittite/Luwian Anatolia involved in the trade of the Late Bronze Age Eastern Mediterranean.

Updating previous R1b-L23* data pertinent to Cyprus [18, 28, 56] reveals that while the presence of R1b-Z2105 in central and west Europe is minimal, conversely, it is informative in present-day Anatolia (10.2 %), Greece (7.0 %), Bulgaria (5.7 %), and Crete (3.1 %). While the emerging R1b-Z2105 substructure [64] has yet to be evaluated at population levels, one relevant sub-haplogroup defined by M589 is illuminating. The majority of Cypriot R1b-Z2105 lineages occur in both the westernmost and easternmost districts, Pafos and Ammochostos, respectively. Notably, of the two districts, the M589 sub-haplogroup occurs only in the east. The absence of M589 lineages in Crete and the Balkans and the presence in Asia Minor are compatible with a record of Late Bronze Age influences from Anatolia rather than from Mycenaean Greeks.

## Conclusions

We report a comprehensive granular Y-chromosome portrait of modern-day Cyprus. Some structural elements are consistent with Neolithic settlements in the central and eastern Mediterranean. Ancient DNA surveys from continental Europe have revealed a subsequent major population replacement within the last five millennia, masking a main part of the pre-Bronze Age genetic substratum [62, 80, 87]. We also detect lineages compatible with Bronze Age communities and subsequent events on Cyprus. The unstructured character of Y-STR lineages within Cyprus, low genetic diversity of Cypriot E-V13, and little genetic differentiation with surrounding populations would support this view.

Support for the perspective that present-day male genetic picture of Cyprus is consistent with an early arrival of pre-historical lineages covered by layers of Y-chromosomes in more recent times is summarized by the following:

(i) Regionally speaking, Cyprus occupies an intermediate position, both geographically and in terms of its Y-chromosome patterning, between Levant, Crete, Italian, and Anatolian/Caucasus populations. Notably, Greek populations show genetic similarities with groups from Balkans. The Greek influence while culturally and linguistically profound only represents a small number of Y-chromosomes common in the Balkans and Carpathian areas.
(ii) The pre-Greek influence is most plausibly encapsulated by the following G2a haplogroups: U5*, PF3147*, L91, L293, P303*, and CTS342. Notably, most of these lineages occur in Anatolian ancient DNA specimens over 8200 years old [53]. In addition, some J2a lineages such as M67, M319, and J2b-M205 may also have contributed to the pre-Greek strata.

Taking a broad perspective, our results are consistent with the model that Cyprus was an early recipient of Levantine-based origin. Interestingly, it apparently remained relatively isolated until experiencing immigration during the Early Bronze Age from Anatolia by early copper metallurgists. This period of isolation and genetic stasis is reminiscent of islands like Sardinia [80] that seem more detached from subsequent vigorous demographic episodes experienced on the mainland.

Although our results report just contemporary patterns and more recent migrations can potentially overwhelm signals of earlier genetic strata, our data approximates perspectives from the archeological record and provides important contextual reference for future ancient DNA studies. The ongoing revolution in ancient genomics [88] heralds the credible opportunity to more comprehensively intersect the cultural and genetic histories of the area and underscores the need for well-attested ancient specimens at population scale sample sizes from Cyprus, Anatolia, the Levant, and southeast Europe.

### Ethics approval and consent to participate

The Cyprus National Bioethics Committee approved the research program and the informed consent process. All donors gave their signed consent for analyzing their DNA anonymously, according to the procedures approved by the Cyprus National Bioethics Committee.

## Additional files

Additional file 1: Table S1. Chronological list of major archaeological strata (adapted from [4]) (DOCX 12 kb) Additional file 2: Figure S1. Geographic sampling locations within the six districts of Cyprus. The Troodos Mountains mainly overlap Pafos, Nicosia, and Limassol districts. (PDF 2414 kb) Additional file 3: Table S2. SNP and STR marker specifications. First sheet presents the genotyped SNPs and indels, method of detection, assigned haplogroup, alternate common names, rs number, chromosomal position (Human Reference GRCh37/hg19 assembly), and nucleotide substitution. Second sheet presents the STR name, repeat structure, and allele range in repeat numbers. (XLSX 19 kb) Additional file 4: Table S3. Y-chromosome haplogroup frequencies and populations included in the PCA of Fig. 3. (XLSX 18 kb) Additional file 5: Figure S2. Spatial frequency distributions of haplogroups J2b-M205 and G2-L293 were generated using Surfer 10 (Golden Software). (PDF 109 kb) Additional file 6: Figure S3. Spatial autocorrelation analysis of Cypriot Y-STR haplotypes (PDF 413 kb) Additional file 7: Figure S4. MDS of the Nei genetic distances between Cypriot Y-STR haplotypes and R script employed to plot the 574 × 574 matrix of genetic distances (PDF 265 kb) Additional file 8: Table S4. SAMOVA results amongst 38 groups of Y-STR haplotypes in Cyprus. FCT: proportion of variance between groups. **p* < 0.05; ****p* < 0.0001 (DOCX 14 kb) Additional file 9: Table S5. Y-chromosome haplogroup specific STR haplotypes for 574 Cypriot samples and haplotype diversity by district (XLSX 101 kb) Additional file 10: Figure S5. E-V13 haplotype network based on 15 Y-STR loci in 46 Greek-Cypriot and 9 Anatolian Greek (Phokaia and Smyrna) samples. Circles represent microsatellite haplotypes, the areas of the circles and sectors are proportional to haplotype frequency (smallest circle corresponds to one individual). Red oval delineates the cluster of haplotypes with DYS437 = 15 repeat allele (PDF 237 kb)

## Abbreviations

*ca.*: circa
SNP: single-nucleotide polymorphism
STR: short tandem repeat or microsatellite
y BP: years before present
