# Supplementary material for: Y-chromosome phylogeographic analysis of the Greek-Cypriot population reveals elements consistent with Neolithic and Bronze Age settlements
Source: Investig Genet. 2016 Feb 11;7:1. doi: 10.1186/s13323-016-0032-8 (PMC4750176; doi:10.1186/s13323-016-0032-8)
Supplement: Additional file 1: Table S1. — Chronological list of major archaeological strata (adapted from [4]) (DOCX 12 kb) [file 13323_2016_32_MOESM1_ESM.docx]

Table S1: Chronological List of Major Archaeological Strata (Adapted from [4])

| Period | Dates BP | Characteristics |
| --- | --- | --- |
| Epipaleolithic | 13,000-11,000 | Hunter-Foragers-Fishers |
| Aceramic Neolithic PPNA | 11,000-10,400 | Initial Neolithic Colonization |
| Aceramic Neolithic PPNB | 10,500-8,800 | Circum-insular Neolithic Sites |
| Late Aceramic Neolithic Khirokita | 9,000-7,200 | Concentrated in S Cyprus |
| Ceramic Neolithic | 7,200-6,000 | First Pottery Production/Levantine Origin? |
| Chalcolithic | 6,000-4,400 | Continuity with Ceramic Neolithic/isolation |
| Early Bronze Age Philia Phase | 4,400-4,250 | Dramatic Shift in Pottery Styles/House Forms/Reappearance of Cattle/West Anatolian Origin? |
| Early Bronze Age/Middle Bronze Age | 4,250-3,700 | Continuity with Philia |
| Late Bronze Age | 3,700-3,100 | International Maritime Trade with Aegean/Anatolian/Levantine Polities/Major Cities in East Cyprus |
