# Supplementary figures and images for: Y-chromosome phylogeographic analysis of the Greek-Cypriot population reveals elements consistent with Neolithic and Bronze Age settlements
Source: Investig Genet. 2016 Feb 11;7:1. doi: 10.1186/s13323-016-0032-8 (PMC4750176; doi:10.1186/s13323-016-0032-8)

Figure S1

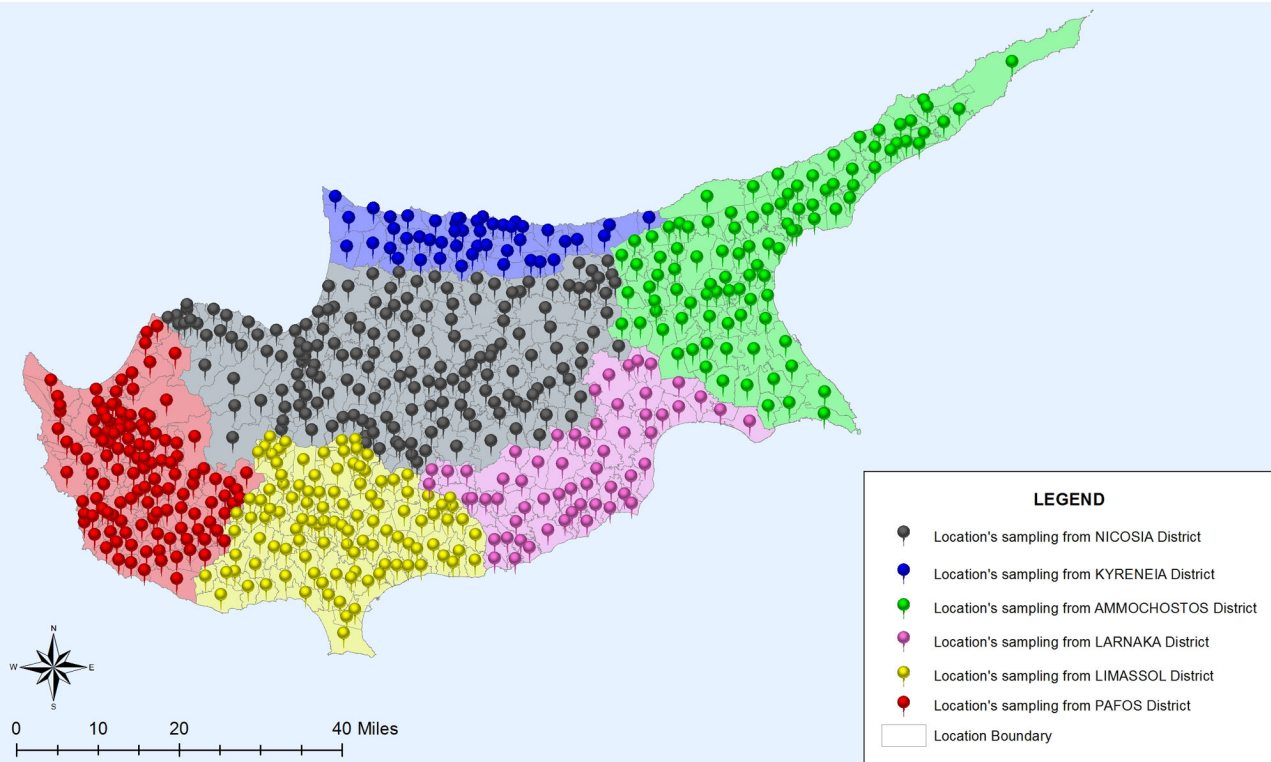

Supplement: Additional file 2: Figure S1. — Geographic sampling locations within the six districts of Cyprus. The Troodos Mountains mainly overlap Pafos, Nicosia, and Limassol districts. (PDF 2414 kb) [file 13323_2016_32_MOESM2_ESM.pdf]

Figure S2

J2b-M205

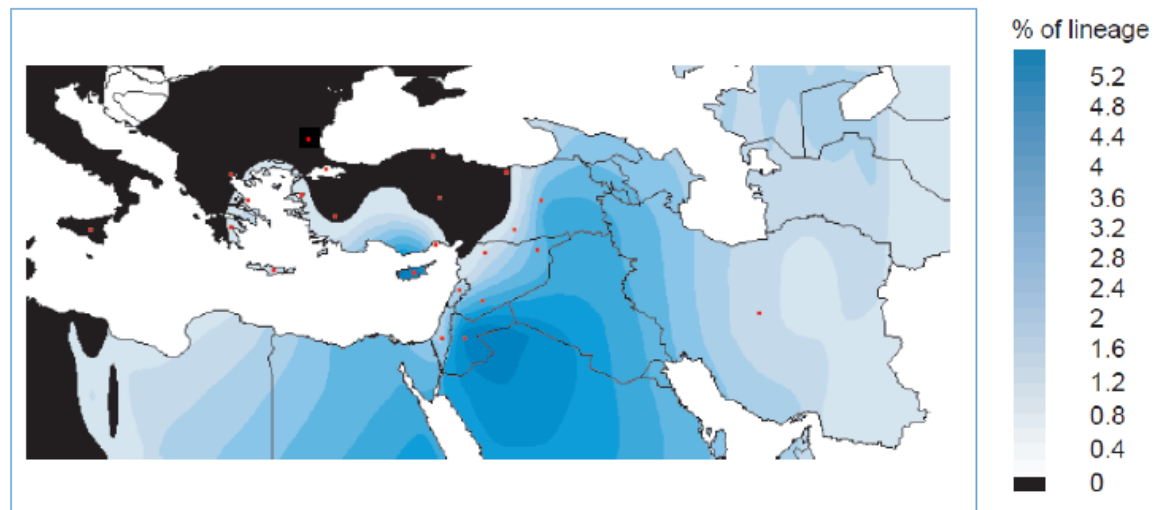

G2a-L293 (xP16)

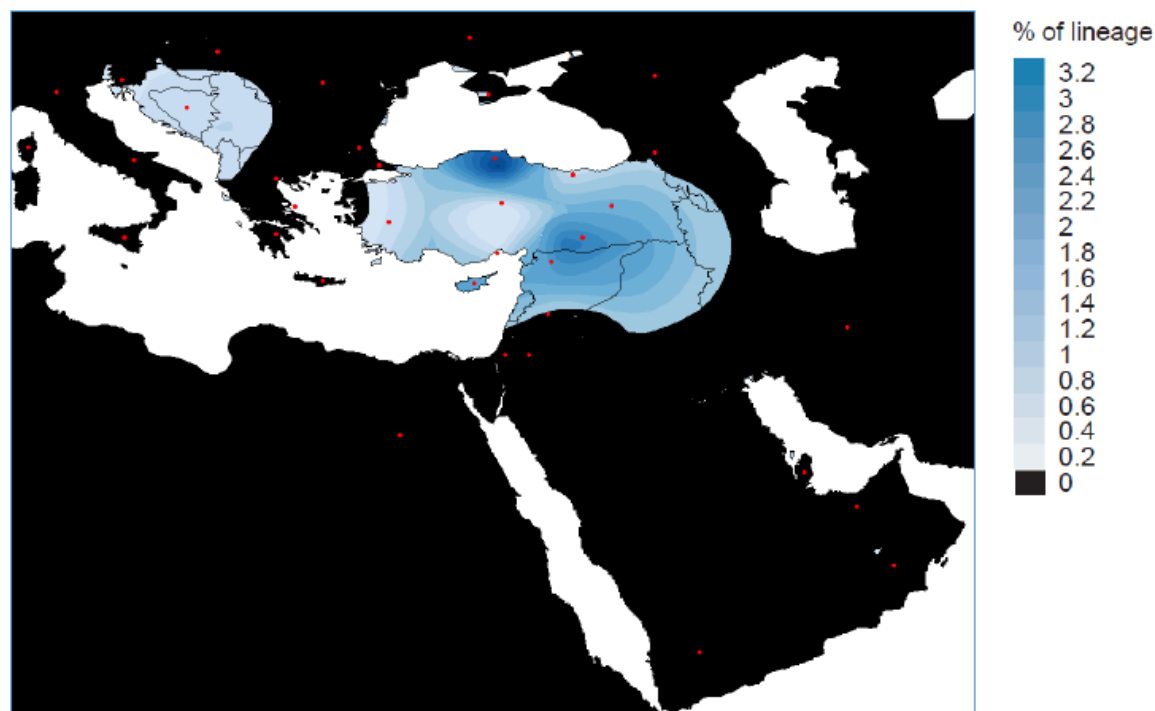

Supplement: Additional file 5: Figure S2. — Spatial frequency distributions of haplogroups J2b-M205 and G2-L293 were generated using Surfer 10 (Golden Software). (PDF 109 kb) [file 13323_2016_32_MOESM5_ESM.pdf]

Figure S3

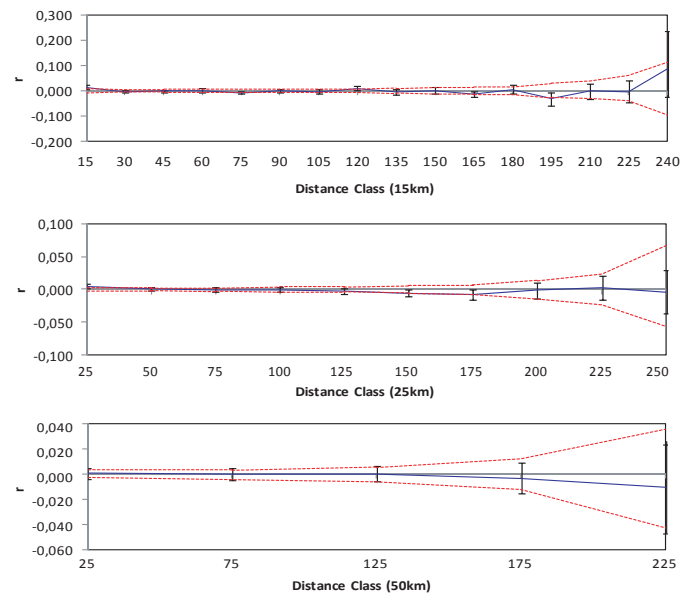

Supplement: Additional file 6: Figure S3. — Spatial autocorrelation analysis of Cypriot Y-STR haplotypes (PDF 413 kb) [file 13323_2016_32_MOESM6_ESM.pdf]

Figure S5

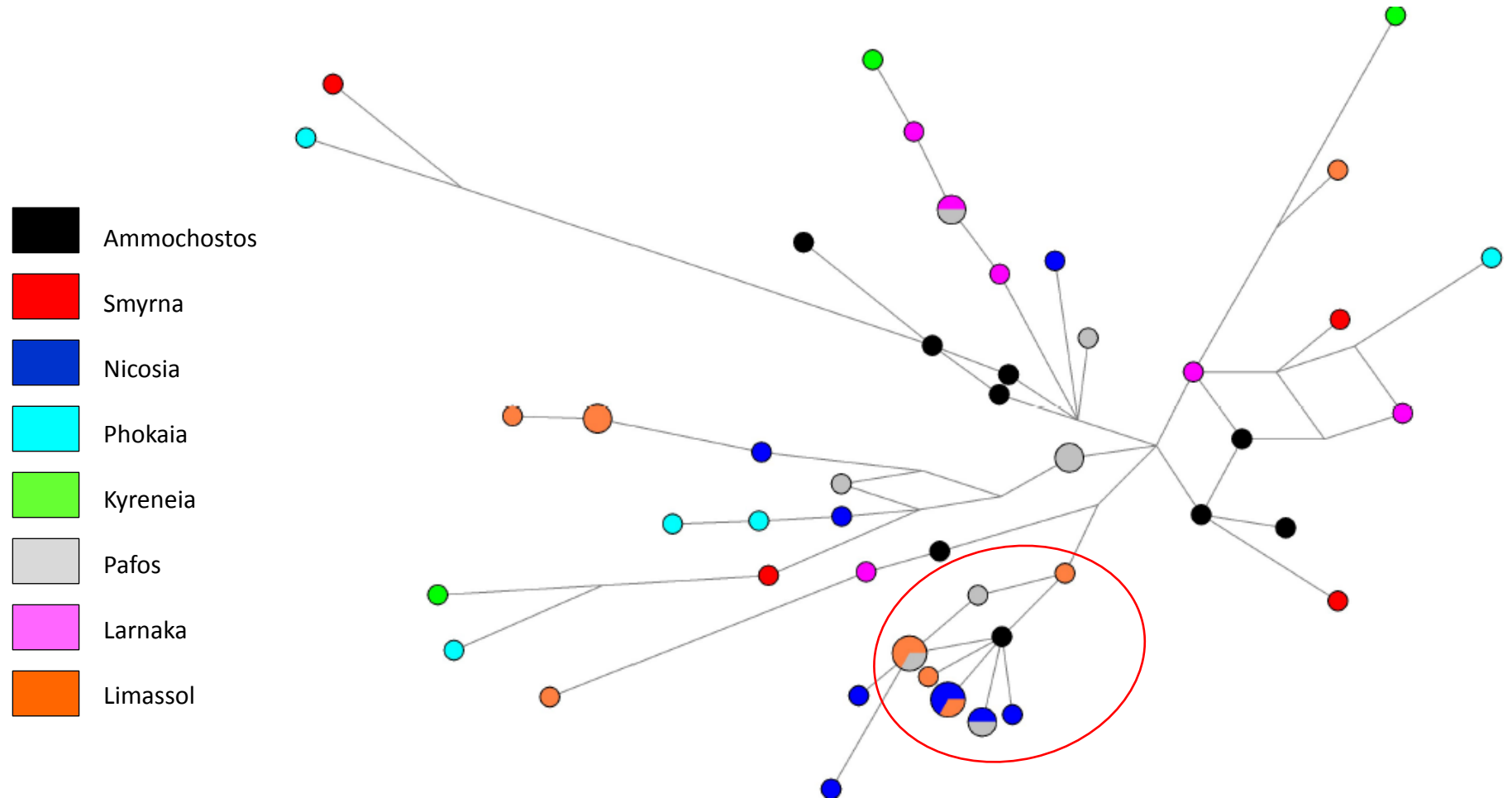

Supplement: Additional file 10: Figure S5. — E-V13 haplotype network based on 15 Y-STR loci in 46 Greek-Cypriot and 9 Anatolian Greek (Phokaia and Smyrna) samples. Circles represent microsatellite haplotypes, the areas of the circles and sectors are proportional to haplotype frequency (smallest circle corresponds to one individual). Red oval delineates the cluster of haplotypes with DYS437 = 15 repeat allele (PDF 237 kb) [file 13323_2016_32_MOESM10_ESM.pdf]
