# Supplementary material for: Y-chromosome phylogeographic analysis of the Greek-Cypriot population reveals elements consistent with Neolithic and Bronze Age settlements
Source: Investig Genet. 2016 Feb 11;7:1. doi: 10.1186/s13323-016-0032-8 (PMC4750176; doi:10.1186/s13323-016-0032-8)
Supplement: Additional file 7: Figure S4. — MDS of the Nei genetic distances between Cypriot Y-STR haplotypes and R script employed to plot the 574 × 574 matrix of genetic distances (PDF 265 kb) [file 13323_2016_32_MOESM7_ESM.pdf]

Figure S4

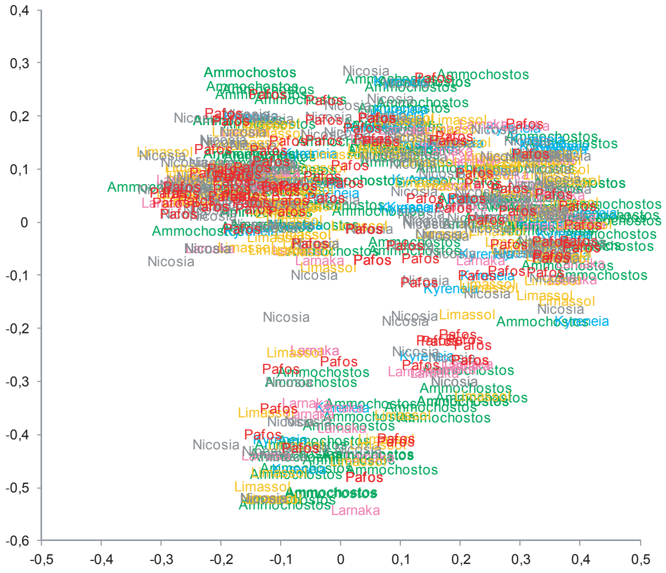


```
### MDS

# importation matrice steph (header=T)

# mise en forme matrice
rownames(steph) <- steph[,1]
steph2 <- steph[,-1]
as.dist(steph2)

# Classical MDS
# N rows (objects) x p columns (variables)
# each row identified by a unique row name

d <- dist(steph2) # euclidean distances between the rows
fit <- cmdscale(d,eig=TRUE, k=2) # k is the number of dim
fit # view results

# plot solution
x <- fit$points[,1]
y <- fit$points[,2]
plot(x, y, xlab="Coordinate 1", ylab="Coordinate 2",
  main="Metric MDS", type="n")
text(x, y, labels = row.names(steph2), cex=.7)
```
