# Supplementary material for: Y-chromosome phylogeographic analysis of the Greek-Cypriot population reveals elements consistent with Neolithic and Bronze Age settlements
Source: Investig Genet. 2016 Feb 11;7:1. doi: 10.1186/s13323-016-0032-8 (PMC4750176; doi:10.1186/s13323-016-0032-8)
Supplement: Additional file 8: Table S4. — SAMOVA results amongst 38 groups of Y-STR haplotypes in Cyprus. FCT: proportion of variance between groups. *p < 0.05; ***p < 0.0001 (DOCX 14 kb) [file 13323_2016_32_MOESM8_ESM.docx]

Table S4. SAMOVA results amongst 38 groups of Y-STR haplotypes in Cyprus. F_CT_ : proportion of variance between groups. *: p<0.05; ***: p<0.0001.

| N° of groups tested for SAMOVA | F_CT_ | Composition of the groups | Geographic distance range (km) within departing groups |
| --- | --- | --- | --- |
| 2 | 20.17* | 37;1 | n/a |
| 3 | 15.87*** | 33;2;3 | [15.38-146.97] |
| 4 | 15.64*** | 33;1;1;3 | [15.38-146.97] |
| 5 | 15.05*** | 33;1;1;2;1 | 15.38 |
| 6 | 14.50*** | 33;1;1;1;1;1 | n/a |
| 7 | 13.60*** | 32;1;1;1;1;1;1 | n/a |
| 8 | 13.28*** | 31;1;1;1;1;1;1;1 | n/a |
| 9 | 13.15*** | 27;1;1;1;1;1;1;1;4 | [15.48-94.7] |
| 10 | 13.06*** | 27;1;1;1;1;1;1;1;1;3 | [15.48-38.64] |
| 15 | 11.17*** | 23;1;…;1;2 | 15.38 |
| 20 | 10.39*** | 14;1;…;1;2;2;3 | [15.48-61.70] |
